# Genetic characterisation of virulence genes associated with adherence, invasion and cytotoxicity in *Campylobacter* spp. isolated from commercial chickens and human clinical cases

**DOI:** 10.4102/ojvr.v85i1.1507

**Published:** 2018-02-15

**Authors:** Samantha Reddy, Oliver T. Zishiri

**Affiliations:** 1 School of Life Sciences, University of KwaZulu-Natal, South Africa

## Abstract

Virulence-associated genes have been recognised and detected in *Campylobacter* species. The majority of them have been proven to be associated with pathogenicity. This study aimed to detect the presence of virulence genes associated with pathogenicity and responsible for invasion, expression of adherence, colonisation and production of the cytolethal distending toxin (cdt) in *Campylobacter jejuni* and *Campylobacter coli*. Commercial chicken faecal samples were randomly sampled from chicken farms within the Durban metropolitan area in South Africa. Furthermore, human clinical *Campylobacter* spp. isolates were randomly sampled from a private pathology laboratory in South Africa. Out of a total of 100 chicken faecal samples, 78% (*n* = 78) were positive for *Campylobacter* growth on modified charcoal cefoperazone deoxycholate and from the random laboratory collection of 100 human clinical isolates, 83% (*n* = 83) demonstrated positive *Campylobacter* spp. growth following culturing methods. These samples were screened for the presence of the following virulence genes: *cadF, hipO, asp, ciaB, dnaJ, pldA, cdtA, cdtB* and *cdtC*. As expected, the *cadF* gene was present in 100% of poultry (*n* = 78) and human clinical isolates (*n* = 83). *Campylobacter jejuni* was the main species detected in both poultry and human clinical isolates, whilst *C. coli* were detected at a significantly lower percentage (*p* < 0.05). Eight per cent of the *C. jejuni* from human clinical isolates had all virulence genes that were investigated. Only one *C. coli* isolate demonstrated the presence of all the virulence genes investigated; however, the *pldA* virulence gene was detected in 100% of the *C. coli* isolates in poultry and a high percentage (71%) in human clinical *C. coli* isolates as well. The detection of *cdt* genes was found at higher frequency in poultry than human clinical isolates. The high prevalence rates of virulence genes detected in poultry and human clinical isolates demonstrate their significance in the pathogenicity of *Campylobacter* species.

## Introduction

Campylobacteriosis is a significant public health concern worldwide with most infections in humans caused by *Campylobacter jejuni* subsp. *jejuni* (from here on referred to as *C. jejuni*) and *Campylobacter coli* (Thakur et al. 2010). Sources of infection of *Campylobacter* spp. in humans include house flies, migratory birds, wildlife, companion animals, livestock, milk, water and other environmental sources (Epps et al. 2013). However, poultry and retail meat products have extensively been reported and implicated as the major sources of infection in human cases of campylobacteriosis (Komba et al. 2013). *Campylobacter jejuni* and *C. coli* have for a long time been the focus of studies within the genus compared to other *Campylobacter* species (Iraola et al. 2014). Research over the years has demonstrated that *C. jejuni* is the primary cause of approximately 80% – 95% of *Campylobacter* infections in human cases and the remaining cause is usually because of *C. coli* (Ragimbeau et al. 2014).

A range of gastrointestinal conditions such as colorectal cancer and inflammatory bowel disease are caused by *Campylobacter* infections. Guillain–Barré syndrome is a rare but serious consequence of *Campylobacter* infections and occurs in 1 out of 1000 cases. Guillain–Barré syndrome is an immune-mediated polyneuropathy of the peripheral nervous system which can result in neuromuscular paralysis (Van den Berg et al. 2014) and Barrett’s oesophagus in humans has also been associated with infection by *Campylobacter* species. Studies have also reported *Campylobacter* spp. in cases of extra-gastrointestinal manifestations such as reactive arthritis, bacteraemia, brain abscesses and lung infections (Broaders, Gahan & Marchesi 2013; Iraola et al. 2014; Nielsen et al. 2010; Swartz 2002). However, the specific role of *Campylobacter* species in the development of all these clinical manifestations remains unclear and further investigation is therefore required (Kaakoush et al. 2015). The epidemiology of *Campylobacter* is complex because of the wide distribution of the bacterium, its genetic variability and its interaction with the host (Khoshbakht et al. 2013). These factors regarding *Campylobacter* spp. have made studying its pathogenicity particularly challenging (Caro-Quintero, Rodriguez-Castaño & Konstantinidis 2009).

Campylobacteriosis is mainly confined to the very young, usually children under the age of 5 years or to elderly patients and patients with immunosuppression (Silva et al. 2011). This presents as a major threat, especially in South Africa because of the extremely high incidence of individuals who are infected with immune-compromising conditions such as HIV and AIDS (Kaakoush et al. 2015), which makes these hosts more susceptible to infection by numerous pathogens including *Campylobacter* spp. Patients infected with *C. jejuni* or *C. coli* or both experience severe watery or bloody diarrhoea, extremely high fevers, major weight loss and severe abdominal cramping which lasts on average for about 6 days. Symptoms usually begin in the period within 24 and 72 h after ingestion of the bacterium depending on the dosage of the organism present in the contaminated food or liquid ingested (Zaidi et al. 2012). Avian species are important reservoirs for the transmission of *Campylobacter* species and their high body temperature provides an optimal environment for the growth of the organism (Noormohamed & Fakhr 2014).

*Campylobacter* are able to colonise the caecum of chicken in extremely high numbers of up to 10^9^ CFU/g of faecal matter, even though this pathogen is present in such high quantities the chicken rarely exhibits symptoms of disease (Thibodeau et al. 2015). The illness in humans can last for a few days, depending on the individual, and the abdominal pain is usually mistaken for appendicitis. Understanding the molecular basis of the virulence-associated genes with disease and knowledge of the genetic diversity amongst the different strains of *Campylobacter* is of paramount importance in the control of diseases and syndromes associated with this organism (Fonseca et al. 2014). Studies have also demonstrated that infections caused by *C. jejuni* and *C. coli* are more common during the summer months of the year (Chansiripornchai & Sasipreeyajan 2009; Fonseca et al. 2014; Garénaux et al. 2008; Hermans et al. 2011). Although *C. coli* is less prevalent than *C. jejuni* in many geographical regions, *C. coli* infections are responsible for as many as 25% of all gastroenteritis clinical cases caused by *Campylobacter* species (Kaakoush et al. 2015).

Gastroenteritis induced by *C. coli* is clinically impossible to differentiate from that by *C. jejuni* because of these organisms being fastidious in nature and their need for nutrient rich-based medium as well as anaerobic conditions for growth. This is the main reason why *Campylobacter* spp. are not applied in routine diagnostic programmes of clinical laboratories in most developing countries (Ghorbanalizadgan et al. 2014). The majority of *Campylobacter* infections are sporadic, and unlike for other food-borne pathogens, huge outbreaks are not very typical. Nevertheless, it is likely that outbreaks or small-case clusters occur far more frequently than previously suggested because of cases of *Campylobacter* infection going unreported and as such the true epidemiological statistics are under-reported (Taboada et al. 2013).

Successful invasion and organisation in host cells depends on the adhesin and fibronectin-binding protein which is the product of the *cadF* gene that plays a significant role during the infection process of *Campylobacter* binding to the extracellular matrix of human intestinal cells (Khoshbakht et al. 2013). The presence of the *ciaB* gene plays a very significant role because of the secretion of a *CiaB* 73 kDa protein which is important for the invasion of epithelial cells as well as colonisation of intestines of avian species, and *CiaB* proteins are also secreted in the presence of poultry serum and mucus (Biswas et al. 2011). The *pldA* gene is also related to cell invasion and is responsible for the synthesis of an outer membrane phospholipase that is important for caecal colonisation (O Cróinín & Backert 2012).

The *dnaJ* virulence gene enables *Campylobacter* species to cope with diverse physiological stresses and is also considered to be a chaperone protein (Chansiripornchai & Sasipreeyajan 2009); the *ciaB, pldA* and *dnaJ* that are recognised as heat shock protein genes are important for caecal colonisation and mutations in these genes limit the ability to achieve this (Jakee et al. 2015). The cytolethal distending toxin (*cdt*) genes, *cdtA, cdtB* and *cdtC*, form polycistronic *cdt* operons that are responsible for the expression of cytotoxins and lethal for host enterocytes (Carvalho et al. 2014). Against this background, this study aimed to isolate and identify *C. jejuni* and *C. coli* from chicken faeces as well and human clinical isolates as well as to determine the prevalence of virulence genes related to adherence, invasion and cytotoxicity.

## Materials and methods

### Ethical considerations

Human and animal studies have been approved by the appropriate ethics committee of the University of KwaZulu-Natal; therefore, they have been performed in accordance with the ethical standards laid down in the 1964 Declaration of Helsinki and its later amendments.

### Origin of samples and processing procedures

One hundred human *Campylobacter* isolates cryopreserved in Brucella broth (Oxoid) with 15% glycerol were randomly sampled from a collection that was received from a private laboratory in Durban, KwaZulu-Natal, during the year 2014 as described by Reddy and Zishiri (2017). In summary, the cultures were revived on modified charcoal cefoperazone deoxycholate (mCCDA) agar (Oxoid, England) containing *Campylobacter*-selective supplement SR0155 (Oxoid, England). A sterile loop was then streaked across the area of inoculation several times to achieve isolated colonies and plates were incubated at 37 °C for 48 h under microaerobic conditions created by CampyGen (Oxoid, UK) gas generating packs in an anaerobic jar.

One hundred chicken faecal samples were randomly sampled between March and September 2016 from commercial farms within the Durban metropolitan area as described by Reddy and Zishiri (2017). Fresh broiler faeces were aseptically collected with sterile swabs then directly inoculated into charcoal broth (Sigma-Aldrich, USA) and then transported to the laboratory for incubation at 37 °C for 48 h, under microaerophilic conditions (5% O_2_, 10% CO_2_ and 85% N_2_) created by CampyGen (Oxoid, UK) gas generating packs in an anaerobic jar.

Following incubation, the faecal samples in charcoal broth (Sigma-Aldrich) were filtered through a 0.65-μm cellulose nitrate filter (Sartorius Stedim Biotech, Germany) onto mCCDA (blood-free agar) (Oxoid, England). Approximately 500 μL of the incubated charcoal broth was evenly distributed over the filter aseptically; once the liquid had been filtered through, forceps were used to aseptically remove the filter. The culture plates were then set in an inverted position in an anaerobic jar containing an atmosphere generation system (CampyGen sachet, Oxoid) and then incubated at 37 °C for 48 h. Following incubation, species identity was confirmed, after Deoxyribonucleic acid (DNA) isolation, by polymerase chain reaction (PCR) targeting of the *hipO* gene specific for *C. jejuni* (Marinou et al. 2012) and the *asp* gene specific for *C. coli* (Al Amri et al. 2013).

Template DNA for PCR was extracted via the conventional boiling method. In summary, characteristic colonies of *Campylobacter* spp. were isolated from plates and suspended in 300 μL TE buffer then vortexed for homogenisation of cells. The suspensions were boiled at 100 °C for 10 min and then immediately cooled on ice. After centrifugation at 14 000 × *g* for 5 min, the supernatants were transferred to a new tube and stored at -20 °C until use in PCR (Datta et al. 2003). A positive *Campylobacter* spp. control was also prepared by isolating DNA from a reference strain of *C. jejuni* ATCC 29428 that was incubated under the same conditions and subjected to the same DNA extraction methods. The Thermo Scientific Nanodrop 2000, UV-Vis spectrophotometer (Wilmington, Delaware, USA) was used to check the concentration and quality of the isolated DNA (Reddy & Zishiri 2017). Following analysis of the extracted DNA, results within the range of 1.8–1.9 at the ratio of 260/280 were regarded as pure DNA and used in PCR. Concentrations of the DNA were also adjusted accordingly with sterile water for subsequent PCR reactions.

### Detection of virulence genes using polymerase chain reaction

The DNA from samples demonstrating positive *Campylobacter* growth was amplified using PCR. In order to differentiate between the species responsible for infection in human clinical cases as well as in poultry, two species-specific genes were used. The *hipO* gene region is the hippuricase gene specific for *C. jejuni* (Marinou et al. 2012) and the *asp* gene region, the aspartokinase gene specific for *C. coli* (Al Amri et al. 2013). The PCR was used to detect nine virulence genes in the total DNA of *Campylobacter* isolates: *cadF, hipO, asp, dnaJ, ciaB, pldA, cdtA, cdtB* and *cdtC*. Polymerase chain reaction primers were sourced from Inqaba Biotechnologies, South Africa. Forward and reverse primers specific for the virulence genes under investigation were designed based on the gene sequence information in the GenBank database and in previously published studies (Al Amri et al. 2007; Chansiripornchai & Sasipreeyajan 2009; Rizal, Kumar & Vidyarthi 2010). Target genes, primer sequences, product sizes and annealing temperatures are presented in Table 1.

**TABLE 1:** Target virulence genes, primer sequences, amplicon sizes and annealing temperatures.

Target gene	Primer sequence (5’–3’)	Product size (bp)	Annealing temperature (°C)	References
*cadF*	F-TTGAAGGTAATTTAGATATG R-CTAATACCTAAAGTTGAAAC	400	43	Chansiripornchai and Sasipreeyajan (2009)
*asp*	F-GGTATGATTTCTACAAAGCGAGA R-ATAAAAGACTATCGTCGCGTG	500	53	Al Amri et al. (2007)
*hipO*	F-GAAGAGGGTTTGGGTGGT R-AGCTAGCTTCGCATAATAACTTG	735	53	Al Amri et al. (2007)
*ciaB*	F-TGCGAGATTTTTCGAGAATG R-TGCCCGCCTTAGAACTTACA	527	54	Chansiripornchai and Sasipreeyajan (2009)
*dnaJ*	F-ATTGATTTTGCTGCGGGTAG R-ATCCGCAAAAGCTTCAAAAA	177	50	Chansiripornchai and Sasipreeyajan (2009)
*pldA*	F-AAGAGTGAGGCGAAATTCCA R-GCAAGATGGCAGGATTATCA	385	46	Chansiripornchai and Sasipreeyajan (2009)
*cdtA*	F-CCTTGTGATGCAAGCAATC R-ACACTCCATTTGCTTTCTG	370	49	Rizal et al. (2010)
*cdtB*	F-GTTAAAATCCCCTGCTATCAACCA R-GTTGGCACTTGGAATTTGCAAGGC	495	51	Rizal et al. (2010)
*cdtC*	F-CGATGAGTTAAAACAAAAAGATA R-TTGGCATTATAGAAAATACAGTT	182	48	Rizal et al. (2010)

Note: Please see the full reference list of the article, Reddy, S. & Zishiri, O.T., 2018, ‘Genetic characterisation of virulence genes associated with adherence, invasion and cytotoxicity in *Campylobacter* spp. isolated from commercial chickens and human clinical cases’, *Onderstepoort Journal of Veterinary Research* 85(1), a1507. https://doi.org/10.4102/ojvr.v85i1.1507, for more information.

Polymerase chain reactions were carried out in the BIO-RAD, T100™ Thermal Cycler (Singapore) for a 25 μL reaction using ThermoScientific DreamTaq Green PCR Master Mix (2X). A total of 12.5 μL DreamTaq Green PCR Master Mix was used with 1.5 μL of each primer of a 10 μM primer concentration, 5 μL template DNA and 4.5 μL nuclease-free water making a total volume of 25 μL. The amplification conditions for *cadF, hipO, asp, dnaJ, ciaB* and *pldA* consisted of an initial denaturalisation at 95 °C for 3 min, 45 cycles at 94 °C for 30 s, specific Tm for each primer for 30 s, and 72 °C for 1 min, followed by a final extension at 72 °C for 5 min. The *cdt* genes (*cdtA, cdtB* and *cdtC*) were run using different amplification conditions according to Rizal et al. (2010). The conditions consisted of an initial denaturation at 94 °C for 15 min, 45 cycles at 94 °C for 1 min, specific Tm for each *cdt* primer for 1 min, and 72 °C for 1 min, followed by a final extension at 72 °C for 7 min. Polymerase chain reaction products were then electrophoresed on a 1.5% agarose gel run at 60 V for 60 min, stained with ethidium bromide and then visualised using the BIO-RAD, ChemiDoc™ MP Imaging System.

### Statistical analyses

Virulence genes detected in *C. jejuni* and *C. coli* were analysed using IBM SPSS Statistics (version 23). Pearson’s correlation analyses were implemented in order to establish the strength and direction of relationships between the individual virulence-associated genes in an effort to ascertain whether the presence of one virulence gene was linked to the presence of the other. Fisher’s exact tests, chi-square tests and logistic regression analysis were implemented to test for significance of whether the presence of virulence genes detected using PCR was determined by whether the isolates emanated from either human clinical cases or chicken faecal samples. In each model that was fitted, the dependent variable was whether a virulence gene was either present or absent (0 = absent; 1 = present) and the explanatory variables were whether the isolates emanated from human clinical cases or chicken faecal samples. The statistics were considered significant when the probability value was less than 0.05 (*p* < 0.05).

## Results

### Prevalence of virulence genes

**FIGURE 1:**
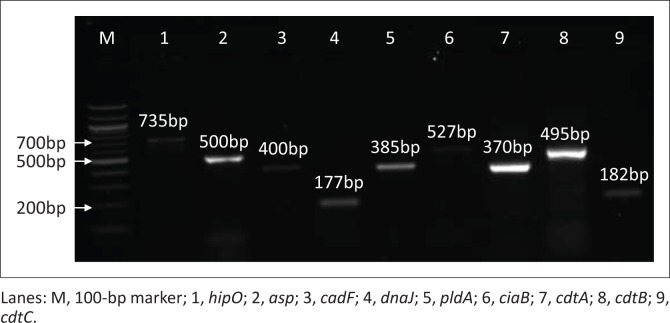
Representative gel picture of species identification and virulence genes investigated from *Campylobacter* spp.

**FIGURE 2:**
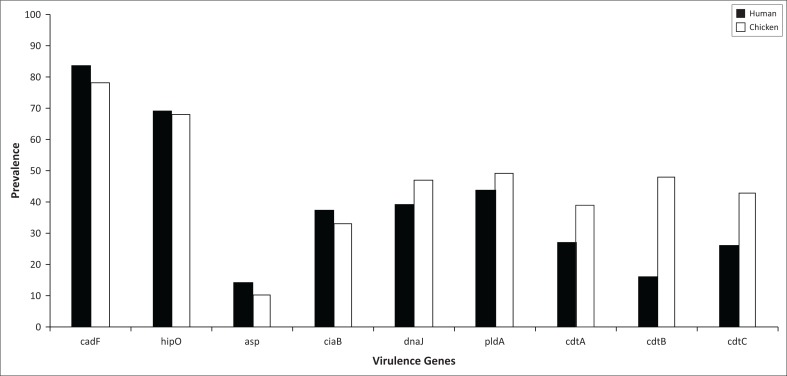
Prevalence of virulence genes in *Campylobacter* spp. in human clinical isolates and chicken faeces.

Of the 100 chicken faecal samples, 78% (*n* = 78) were presumptive for *Campylobacter* spp. growth on mCCDA and from the collection of a 100 human clinical isolates, 83% (*n* = 83) demonstrated positive *Campylobacter* spp. growth following culturing methods. Virulence genes investigated in this study are shown in Figure 1 and species differentiation was confirmed by detection of *hipO* (lane 1: 735 bp) and *asp* gene (lane 2: 500 bp). *Campylobacter jejuni* is the only species known to have the hippuricase (*hipO*) gene because it has not been detected in any other *Campylobacter* species. Furthermore, the aspartokinase (*asp*) gene is specific only in *C. coli*. All isolates (100%), irrespective of the species, were positive for the *cadF* gene based on PCR detection of a 400 bp amplicon (lane 3). This gene encodes *Campylobacter* species adhesion to fibronectin that is an important virulence factor for colonisation of epithelial cells. The *dnaJ* gene is depicted in lane 4 at 177 bp. Lane 5 depicts the *pldA* virulence gene with an amplicon of 385 bp. Lane 6 is the *ciaB* gene at 527 bp, lane 7 is the *cdtA* gene (370 bp), lane 8 is the *cdtB* gene (495 bp) that is a catalytic subunit in the CDT cluster and lastly lane 9 is the *cdtC* gene (182 bp), and *cdtA* and *cdtC* are binding proteins responsible for delivering *cdtB* into target cells. The number of isolates that were positive of each virulence gene is depicted in Figure 2.

**FIGURE 3:**
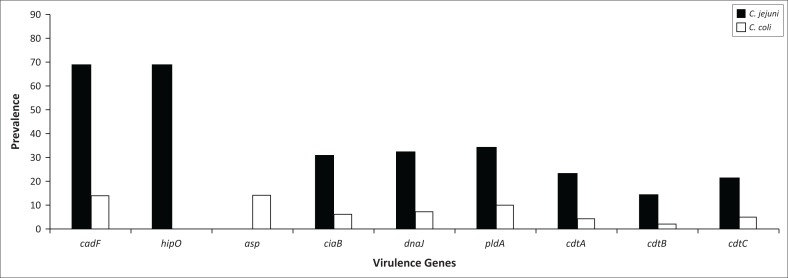
Prevalence of virulence genes in *Campylobacter jejuni* and *Campylobacter coli* isolated from human clinical isolates.

Results in Figure 3 indicate that *C. jejuni* is responsible for the majority of infection (83%; *n* = 69) in human gastroenteritis cases compared to the low detection of *C. coli* found only in 17% (*n* = 14) of cases. Of the *C. jejuni* isolated from human clinical cases, 45%, 46%, 49%, 33%, 20% and 30% were positive for *ciaB, dnaJ, pldA, cdtA, cdtB* and *cdtC*, respectively, and *C. coli* revealed 43%, 50%, 71%, 29%, 14% and 36%, respectively.

**FIGURE 4:**
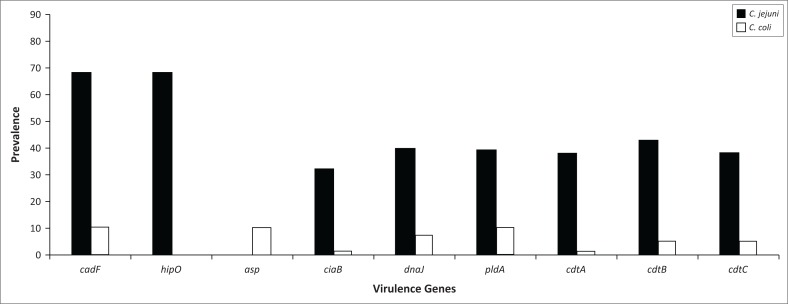
Prevalence of virulence genes in *Campylobacter jejuni* and *Campylobacter coli* isolated from chicken faeces.

The isolates from chicken faeces (Figure 4) demonstrated a higher percentage of *C. jejuni* (87%) (*n* = 68) present as compared to only 13% (*n* = 10) of *C. coli*. The poultry isolates for *C. jejuni* demonstrated 47%, 59%, 57%, 56%, 63% and 56% for the presence of *ciaB, dnaJ, pldA, cdtA, cdtB* and *cdtC*, respectively, and *C. coli* isolates revealed 10%, 70%, 100%, 10%, 50% and 50% for the presence of *ciaB, dnaJ, pldA, cdtA, cdtB* and *cdtC*, respectively.

### Statistical analyses

Pearson’s correlations (Table 2) demonstrated significant (*p* < 0.05) positive correlations between some virulence genes. The *asp* gene that identifies *C. coli* was the only gene which was not statistically significantly (*p* > 0.05) correlated with some of the virulence genes such as *ciaB, dnaJ* and all the *cdt* genes screened. Generally, most of the correlations amongst the virulence genes were statistically significant (*p* < 0.05), as depicted in Table 2. The presence of the *dnaJ* and *cdt* genes was positively correlated (*p* < 0.05). Furthermore, the presence of the *hipO* that confirms the presence of *C. jejuni* was positively correlated (*p* < 0.05) with the presence of the *dnaJ* gene and the *cdt* genes. There was also evidence of a positive correlation (*p* < 0.05) between the *ciaB* gene, *dnaJ, pldA* and *cdt* genes.

**TABLE 2:** Comparison of Pearson’s correlations for virulence genes detected in *Campylobacter* species from human clinical isolates and chicken faeces.

Genes	Statistical tests	*cadF*	*hipO*	*asp*	*ciaB*	*dnaJ*	*pldA*	*cdtA*	*cdtB*	*cdtC*
*cadF*	Pearson’s correlation	1	0.630**	0.141	0.286**	0.342**	0.369**	0.272**	0.266**	0.282**
	Sig. (2-tailed)	-	0.000	0.058	0.000	0.000	0.000	0.000	0.000	0.000
*hipO*	Pearson’s correlation	0.630**	1	−0.680**	0.270**	0.185*	0.076	0.300**	0.235**	0.185*
	Sig. (2-tailed)	0.000	-	0.000	0.000	0.012	0.306	0.000	0.001	0.012
*asp*	Pearson’s correlation	0.141	−0.680**	1	−0.074	0.087	0.251**	−0.125	−0.049	0.030
	Sig. (2-tailed)	0.058	0.000	-	0.318	0.246	0.001	0.092	0.512	0.686
*ciaB*	Pearson’s correlation	0.286**	0.270**	−0.074	1	0.157*	0.209**	0.484**	0.340**	0.406**
	Sig. (2-tailed)	0.000	0.000	0.318	-	0.035	0.005	0.000	0.000	0.000
*dnaJ*	Pearson’s correlation	0.342**	0.185*	0.087	0.157*	1	0.442**	0.339**	0.340**	0.327**
	Sig. (2-tailed)	0.000	0.012	0.246	0.035	-	0.000	0.000	0.000	0.000
*pldA*	Pearson’s correlation	0.369**	0.076	0.251**	0.209**	0.442**	1	0.349**	0.329**	0.334**
	Sig. (2-tailed)	0.000	0.306	0.001	0.005	0.000	-	0.000	0.000	0.000
*cdtA*	Pearson’s correlation	0.272**	0.300**	−0.125	0.484**	0.339**	0.349**	1	0.354**	0.400**
	Sig. (2-tailed)	0.000	0.000	0.092	0.000	0.000	0.000	-	0.000	0.000
*cdtB*	Pearson’s correlation	0.266**	0.235**	−0.049	0.340**	0.340**	0.329**	0.354**	1	0.421**
	Sig. (2-tailed)	0.000	0.001	0.512	0.000	0.000	0.000	0.000	-	0.000
*cdtC*	Pearson’s correlation	0.282**	0.185*	0.030	0.406**	0.327**	0.334**	0.400**	0.421**	1
	Sig. (2-tailed)	0.000	0.012	0.686	0.000	0.000	0.000	0.000	0.000	-

* Correlation is significant at the 0.05 level (2-tailed)

** correlation is significant at the 0.01 level (2-tailed).

There were statistically significant (*p* < 0.05) relationships between the *cadF, hipO, dnaJ, pldA, cdtA, cdtB* and *cdtC* genes because of their presence in *Campylobacter* human clinical cases and chicken faeces investigated (Table 3). The virulence genes *asp* and *ciaB* were not statistically significant from the results observed because the results demonstrated a *p* > 0.05 for chi-square and Fisher’s exact statistical tests when compared to the other genes investigated.

**TABLE 3:** Chi-square test and Fisher’s exact test for virulence genes investigated.

Statistical tests	Asymptotic significance (2-sided)								
	*cadF*	*hipO*	*asp*	*ciaB*	*dnaJ*	*pldA*	*cdtA*	*cdtB*	*cdtC*
Pearson’s chi-square test	0.011	0.030	0.720	0.654	0.014	0.034	0.004	0.000	0.000
Fisher’s exact test	0.011	0.038	0.827	0.760	0.017	0.038	0.005	0.000	0.000

Logistic regression analyses were conducted to predict the presence of virulence genes in chicken faeces and human clinical isolates using the source of the isolates as a predictor (Table 4). A test of the full model against a constant only model indicated that genes *ciaB, dnaJ* and *pldA* were not statistically significant (*p* > 0.05). The Wald criterion demonstrated that *cdtA, cdtB* and *cdtC* virulence genes made a significant contribution to prediction of the presence of these genes in human clinical isolates and poultry with *p* = 0.025, < 0.001 and 0.003, respectively (*p* < 0.05).

**TABLE 4:** Logistic regression analysis results demonstrating the significance of virulence genes found in human clinical isolates and chicken faeces.

Virulence genes	-2 Log likelihood	*B*	*SE*	Wald *p*-value	*OR*	95% CI
*ciaB*	220.362	0.092	0.318	0.771	1.097	0.588–2.047
*dnaJ*	219.587	−0.537	0.319	0.093	0.585	0.313–1.093
*pldA*	217.706	−0.404	0.321	0.209	0.668	0.356–1.253
*cdtA*	212.844	−0.730	0.326	0.025	0.482	0.255–0.913
*cdtB*	185.315	−1.902	0.363	0.000	0.149	0.073–0.304
*cdtC*	210.508	−0.991	0.328	0.003	0.371	0.195–0.707

## Discussion

*Campylobacter jejuni* and *Campylobacter coli* are the most prevalent *Campylobacter* spp. that are responsible for gastroenteritis infections in humans. The pathogenicity of *Campylobacter* spp. depends on the virulence factors that differ amongst strains of different origin. Several virulence-associated genes have been recognised and detected in *Campylobacter*, and the majority of them are associated with pathogenicity. The information regarding *Campylobacter* virulence genes and genotypes of *Campylobacter* spp. is unclear in developing countries such as South Africa; therefore, research is essential to characterise pathogenic markers in this organism and to, furthermore, initiate subsequent preparation of strategies for its proper control and prevention.

All isolates that were presumptively identified as *Campylobacter*, irrespective of the bacterial species, were positive for the presence of the *cadF* gene, which facilitates adherence to fibronectin in contact regions (Flanagan et al. 2009). These findings are in concordance with earlier studies with regard to the presence of the *cadF* gene in *Campylobacter* spp. isolated from human as well as chicken (Al Amri et al. 2007; Datta et al. 2003; Rozynek et al. 2005). The presence of gene products from *cadF* (*Campylobacter* adhesin to fibronectin) has clearly demonstrated involvement in *Campylobacter* colonisation and this has been shown by *in vivo* colonisation using a chicken model (Thibodeau et al. 2015). This gene encodes a protein that interacts with a host extracellular matrix protein fibronectin, and is required for *Campylobacter* adherence to and colonisation of the host cell surface (Ghorbanalizadgan et al. 2014). The high prevalence (100%) of the *cadF* gene in the present study demonstrates that many isolates originating from poultry faeces have pathogenic potential properties for humans. The ubiquitous existence of the highly conserved *cadF* gene in 100% of *Campylobacter* spp. was previously reported by Konkel et al. (1999) and was subsequently used by other investigators for successful detection of *Campylobacter* spp. (Rizal et al. 2010; Wieczorek, Szewczyk & Osek 2012).

The hippuricase gene (*hipO*) is specific for *C. jejuni* and is not detected in any other *Campylobacter* species. The Pearson’s correlation analyses demonstrated that there was a significant (*p* < 0.05) strong positive correlation (63%) between the *cadF* and the *hipO* genes. Our study utilised the positive PCR amplification of *asp* gene in order to identify which samples belonged to the species *C. coli*. Furthermore, the correlation analysis demonstrated that the presence of the *asp* gene was negatively correlated (-0.63%) to the presence of the *hipO* gene (*p* < 0.05). This is in agreement with other studies because *C. coli* is not as prevalent as *C. jejuni* as the cause of infection in humans (Feodoroff et al. 2011; Khoshbakht et al. 2013; Samie et al. 2007).

Many factors play a significant role in the varying isolation or recovery rates of *Campylobacter* species that include whether the samples are fresh or frozen, the type of sampling procedure used and the isolation protocol followed as well as the time of year for collection of samples. The *ciaB* (*Campylobacter* invasive antigen B) gene is known to be involved in the translocation of *Campylobacter* into host cells for the purpose of host cell invasion and also plays a significant role in caecal colonisation in chicken (O Cróinín & Backert 2012). The *ciaB* gene was detected in 45% and 42% in human and chicken samples, respectively, and had a significant low positive correlation (15.7%) with the *dnaJ* gene (*p* < 0.05). With regard to the *dnaJ* gene that enables *Campylobacter* species to cope with diverse physiological stresses, there was a significant positive correlation when there was presence of the *hipO* and *ciaB* gene found in samples (*p* < 0.05).

Another important factor for colonisation of *Campylobacter* species in the intestine of chickens is the *pldA* gene (an outer membrane phospholipase A) that encodes proteins associated with increased bacterial invasion on cultured epithelial cells (Ghorbanalizadgan et al. 2014). The distribution of this gene was dissimilar amongst the two species investigated as *C. jejuni* showed a higher presence of this gene compared to *C. coli* in both human and chicken isolates. The percentage of *pld*A in chicken and human isolates was 53% and 63%, respectively.

There is an increase ranging from 88% to 100% in the presence of the *pldA* gene in *C. jejuni* isolates from chicken with age of broilers as a major contributing factor (Datta, Niwa & Itoh 2009). The collection of samples recovered from broilers at the end of the breeding period increases the chances of poultry serovars having better colonisation and making them more virulent for their other hosts. The results from this study indicate that *Campylobacter* spp. recovered from chicken faeces may have potential virulence consequences in humans. The study also demonstrated that *C. jejuni* and *C. coli* in chicken faecal samples from the slaughterhouse are indicative of a public health hazard because of the spread of this emerging food-borne pathogen. The contamination of carcasses may occur from intestinal contents during slaughtering and or post-slaughtering processes and if these products are not cleaned, stored or cooked properly, this could lead to outbreaks of infection in the public.

Damage to nuclear DNA resulting in the inhibition of the cell cycle in G2 or M phase is the cytopathic effect of the cytotoxin released by *cdtA, cdtB* and *cdtC* (Carvalho et al. 2014). The low percentage of the *cdtB* gene in humans found in this study (19%) is in agreement with a study conducted in India (Rizal et al. 2010), which reported a prevalence of 28%. However, chickens also demonstrated a low percentage of this gene (20%) in contrast to this study which found 62% that could be because of genetic factors or variation in the isolates from different geographic areas. Translocation of *cdtB* to the nucleus of cells induces the genotoxic effects on host DNA; triggering DNA repair mechanisms that could lead to cell cycle arrest and eventually cause cell death (Ge et al. 2008). In addition, it has also been suggested that the CDT cluster could also play a role in adhesion and invasion of the pathogen in a host (Ge et al. 2008). Cell invasion could result in cellular injury, leading to reduced absorptive capacity of the intestine, whereas *cdt* production is important for interleukin-8 release by intestinal cells *in vitro* which plays an important role in the host mucosal inflammatory response caused by *Campylobacter* species (Hermans et al. 2011).

Cell invasion of epithelial cells and *cdt* production are important bacterial virulence mechanisms that play a significant role for inducing enterocolitis. The presence of a single *cdt* gene does not have any effect on the bacterium virulence; however, if all three *cdt* genes are present together in a cluster, then they are responsible for the release of a functional cytotoxin (Lapierre et al. 2016). Our study revealed that 10 of the human clinical isolates had all three *cdt* genes compared to 18 of the chicken isolates. The low percentage of *cdt* genes needs to be compared with cytolethal distending toxin production levels and tested on a variety of different cell lines in order to study the phenotypic characteristics of the isolates for a better understanding (Wieczorek, Szewczyk & Osek 2012).

## Conclusion

The current study revealed that isolates of *C. jejuni* and *C. coli* from chicken faeces and human clinical cases possess a variety of different virulence genes associated with vital processes such as invasion, from the genes investigated in this study: *cadF, ciaB* and *pldA* play significant roles in successful invasion within a host. Expression of adherence in host cells depends on the adhesin and fibronectin-binding protein which is the product of the *cadF* gene and this gene was present at 100% in both chicken faecal samples (*n* = 78) and human clinical isolates (*n* = 83). Colonisation within a host also plays an important role for *Campylobacter* survival and *ciaB, pldA* and *dnaJ* are important for caecal colonisation in chickens; however, mutations in these genes can limit the ability to achieve this. The production of cytolethal distending toxins *cdtA, cdtB* and *cdtC* forms a polycistronic *cdt* operon that is responsible for the expression of cytotoxins, from these genes, and lethal for host enterocytes. Findings from this study demonstrate the pathogenic potential of the isolates recovered and this is a major public health concern. This necessitates the need for proper preventive measures to prevent contamination of food with *Campylobacter* spp. at the food production level in South Africa. This is one of the first studies emanating from South Africa in which the virulence genes in *Campylobacter* spp. have been screened in both human and animal samples and is therefore a good foundation for more intensive studies.
